# A Random Screen Using a Novel Reporter Assay System Reveals a Set of Sequences That Are Preferred as the TATA or TATA-Like Elements in the *CYC1* Promoter of *Saccharomyces cerevisiae*

**DOI:** 10.1371/journal.pone.0129357

**Published:** 2015-06-05

**Authors:** Kiyoshi Watanabe, Makoto Yabe, Koji Kasahara, Tetsuro Kokubo

**Affiliations:** Molecular and Cellular Biology Laboratory, Graduate School of Medical Life Science, Yokohama City University, Yokohama, Kanagawa, Japan; Univeristy of California Riverside, UNITED STATES

## Abstract

In *Saccharomyces cerevisiae*, the core promoters of class II genes contain either TATA or TATA-like elements to direct accurate transcriptional initiation. Genome-wide analyses show that the consensus sequence of the TATA element is TATAWAWR (8 bp), whereas TATA-like elements carry one or two mismatches to this consensus. The fact that several functionally distinct TATA sequences have been identified indicates that these elements may function, at least to some extent, in a gene-specific manner. The purpose of the present study was to identify functional TATA sequences enriched in one particular core promoter and compare them with the TATA or TATA-like elements that serve as the pre-initiation complex (PIC) assembly sites on the yeast genome. For this purpose, we conducted a randomized screen of the TATA element in the *CYC1* promoter by using a novel reporter assay system and identified several hundreds of unique sequences that were tentatively classified into nine groups. The results indicated that the 7 bp TATA element (i.e., TATAWAD) and several sets of TATA-like sequences are preferred specifically by this promoter. Furthermore, we find that the most frequently isolated TATA-like sequence, i.e., TATTTAAA, is actually utilized as a functional core promoter element for the endogenous genes, e.g., *ADE5*,*7* and *ADE6*. Collectively, these results indicate that the sequence requirements for the functional TATA or TATA-like elements in one particular core promoter are not as stringent. However, the variation of these sequences differs significantly from that of the PIC assembly sites on the genome, presumably depending on promoter structures and reflecting the gene-specific function of these sequences.

## Introduction

In eukaryotes, transcription is highly regulated by the physical interactions between transcriptional regulators that bind to the upstream activating sequence (UAS in budding yeast) or the enhancer (in higher eukaryote) and a cohort of transcription factors that assemble on the core promoter to form the pre-initiation complex (PIC) (reviewed in [1, 2]). The core promoter region contains a variety of functionally distinct small DNA segments (termed “core promoter elements”) such as the TATA element, BRE^u/d^, Inr/sINR, TCT motif, DPE, MTE, bridge element, DCE, and XCPE1/2 (reviewed in [3–5]). Most of these elements are recognized by general transcription factors (GTFs), including TFIIB or TFIID [6]. However, the effect of such variation in the core promoter elements on the transcriptional regulation of eukaryotic class II genes remains unclear.

The existence of functional compatibility between the UAS/enhancer and the core promoter in various organisms has been suggested [7–10]. In budding yeast, for instance, the UAS of *RPS5* can activate the *ADH1* or *CUP1* core promoter, whereas the UAS of the latter two genes cannot activate the *RPS5* core promoter [11]. In *Drosophila*, a recent genome-wide study demonstrated that the TCT motif-containing core promoter and the TATA-Inr-MTE-DPE-containing core promoter are activated by different sets of enhancers [12]. Caudal, a key regulator of the *Hox* genes in *Drosophila*, can activate the DPE-containing core promoter but cannot activate the BRE^u^-TATA-containing core promoter [13]. Similarly, Dorsal, a key regulator of the dorsal-ventral gene regulatory network in *Drosophila*, preferentially activates the DPE-containing core promoter over the TATA-containing core promoter [14]. The latter two observations might be accounted for by a recent finding that the DPE-containing core promoter is recognized not only by TBP-containing TFIID [15], but also by TRF2 (TBP-related factor 2)-containing complexes [16]. Furthermore, recent studies show that transcription of the TCT motif-containing core promoter depends on TRF2 but not on TBP/TFIID [17, 18]. Therefore, it is likely that the core transcriptional machinery recruited by a given transcriptional regulator could become fully competent only when it is recruited to the core promoter with an appropriate structure.

In budding yeast, no obvious sequence motifs in core promoter elements other than the TATA element (i.e., TATAWAWR) [19, 20] and/or the Inr element [21] have been identified [19, 20]. Although TATA function appears to be highly conserved among eukaryotes, Inr function may differ between budding yeast and metazoans; for instance, it serves as a recognition site for the TAF1-TAF2-TBP sub-complex of TFIID in the latter [22], whereas it represents the preferred initiation site for pol II in the former [20, 23, 24]. Furthermore, there are no known TBP homologues in budding yeast, such as TRFs, although the UAS still shows core promoter specificity as mentioned above [11, 25, 26]. A recent genome-wide study using the ChIP-exo method demonstrated that nearly all TATA-less promoters (i.e., corresponding to approximately 90% of all class II genes in budding yeast) contain sequences with two or less mismatches to the TATA consensus (i.e., TATAWAWR) at the PIC assembly sites [27]. This result is consistent with our previous observation that multiple AT-rich sequences could function as a core promoter element individually in the TATA-less *RPS5* core promoter [20]. Therefore, yeast class II promoters can be divided into two subclasses: one includes promoters containing TATAWAWR and the other includes those containing similar but slightly different derivatives of TATAWAWR. These two subclasses may correspond to the SAGA- and TFIID-dominated promoters, respectively [28]. These accumulating evidences suggest that budding yeast could provide a simple model system to investigate the core promoter specificity of the UAS/enhancer, since there are few types of core promoter elements (e.g., TATAWAWR and its variants) in this organism.

As reported previously (reviewed in [7]), transcriptional regulators could function selectively with specific TATA (or TATA-like) sequences. For instance, the *HIS3* core promoter contains two different types of TATA elements, namely, T_C_ and T_R_, and only the latter can be activated by Gcn4p and Gal4p [23]. Intriguingly, three derivatives of T_R_ can be activated by Gcn4p but not by Gal4p [29]. Similar functional discrimination between different TATA sequences by transcriptional regulators is observed in mammals [30, 31], indicating that the regulatory system based on sequence-specific TATA function may be widely exploited among eukaryotes from yeast to mammals. In this regard, the *CYC1* promoter is particularly interesting as it contains two structurally similar but functionally distinct TATA elements: TATAβ (ATATATA*T*AT) and TATAα (TATATA*A*AA) [32]. These two TATA sequences differ only at one position (italicized base *T* vs. *A* as shown above) when comparing the underlined regions that match to the consensus TATAWAWR. However, how such a subtle difference is recognized properly by the core transcriptional machineries to execute two different functions remains obscure.

The original aim of the present study was to identify the sequence(s) that are functionally identical to TATAβ or TATAα by a randomized screen, and delineate molecular determinant(s) between these two types of TATA elements. For this purpose, we developed a novel reporter assay system that measures the amounts of polyphosphate (polyP) accumulated in yeast cells. This method is simple and cost-effective, as it uses a spectrophotometer instead of an expensive NMR machine for the measurement [33, 34]. However, the original aim to isolate two functionally distinct TATA sequences was unsuccessful, since any functional differences between TATAβ and TATAα could not be recapitulated at the reporter gene locus. However, we found that TATAα acquires Taf1p/TFIID-dependency at this locus, indicating that the recognition mode of this element by the core transcription machineries might be altered from a Taf1p-independent (e.g., SAGA) to a Taf1p-dependent (e.g., TFIID) manner. A similar screen was hitherto conducted only for the Taf1p-independent TATA element, i.e., T_R_ of the *HIS3* promoter [35]. Therefore, we conducted a randomized screen to identify functional derivatives of the Taf1p-dependent TATAα element of the *CYC1* promoter. More than 600 independent sequences were isolated and classified tentatively into nine distinct groups. Comparison of these sequences with functional derivatives of T_R_ [35] and/or TATA or TATA-like elements identified as the PIC assembly sites in many other genes [27] indicated that functional sequences may vary significantly among genes, presumably depending on promoter structures. Furthermore, the high saturation level of our screen enabled, for the first time, the identification of a possible range of functionally competent sequences in one particular promoter. Finally, we showed that the TATTTAAA sequence isolated most frequently by the screen is used as a core promoter element for the endogenous genes. These results imply that the experimental approaches developed in this work may be effective to identify a large number of TATA or TATA-like sequences that can function specifically in a subset of genes in budding yeast, and to investigate the mechanisms underlying the gene-specific function of these sequences.

## Materials and Methods

### Yeast strains

Standard techniques were used for yeast growth and transformation [36]. Yeast strains used in this study are listed in S1 Table. Oligonucleotide sequences used for strain construction are listed in S2 Table.

**Fig 1:**
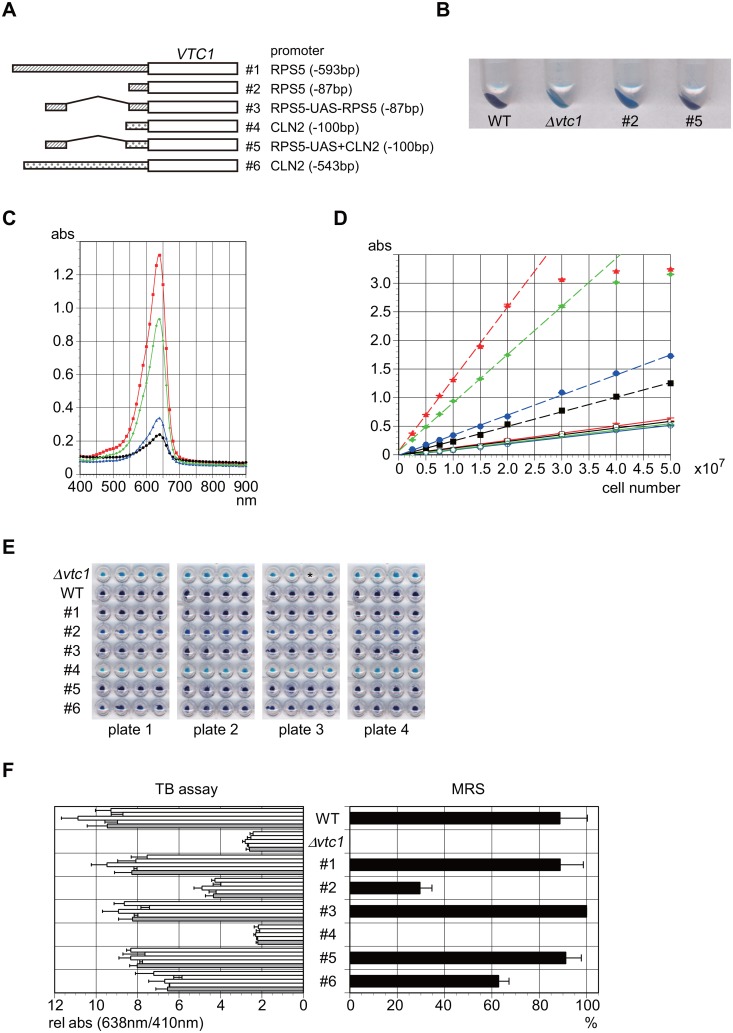
A novel reporter assay system measuring gene expression in yeast cells. **A**. Schematic diagram of the reporter constructs under the control of six different promoters, as described previously [33]. The strains tested in this figure were constructed from the wild-type strain (WT; BY4741) by replacing its endogenous *VTC1* promoter with one of these #1–6 promoters. The promoter activities (i.e., polyphosphate accumulation) of these strains were measured by MRS (magnetic resonance spectroscopy) or MRI (magnetic resonance imaging) in our previous study [33]. **B**. Photograph of the four yeast strains taken after staining with toluidine blue (TB) dye. These strains are the wild-type (WT; BY4741), *Δvtc1* (Y00212), and those carrying #2 (YTK6353) or #5 (YTK6362) promoter constructs. **C**. Absorption spectra of the set of four strains described in **B**. The spectrum of each strain is shown in a different color; WT (red), *Δvtc1* (black), #2 (blue), or #5 (green). Measurements were performed using 1x10^7^ cells of each strain. **D**. Absorption at 638 nm (broken line) or 410 nm (solid line) of the set of four strains described in **B** is plotted as a function of the number of cells, as indicated in the horizontal axis. Each strain is represented by the same color described in **C**. **E**. Photograph of the eight yeast strains (WT, *Δvtc1*, and #1–6 as described in **A**), which were cultured and processed in a 96-well microplate format, taken after the TB staining. Each strain was inoculated into four wells of each plate. Here, the four plates were processed in parallel for TB staining. The third well from the left at the top row of the plate 3 (marked with an asterisk) is empty because the *Δvtc1* strain was inoculated in this well but was lost accidentally during the process. **F**. The absorption at 638 nm normalized by that at 410 nm for each strain was measured and summarized in the left panel. The average of the four wells in each plate or that of the four plates is shown as white or gray bars, respectively, along with the standard deviation. The amounts of polyphosphate in each strain were measured by MRS previously [33] and cited in the right panel as a reference.

All strains used in Fig 1 were obtained from Euroscarf (BY4741 and Y00212) or described previously (YTK6352, YTK6353, YTK6356, YTK6359, YTK6362, and YTK6365) [33].

All strains used in other Figs were generated from BY4741 by a fusion PCR-based method [37, 38], as described below. To create YTK7548, the four sub-fragments containing *VTC1* [-320 –-11 bp] (TK7872-TK7873/BY4741), *His3MX6* (TK8297-TK10263/pFA6a-His3MX6), *CYC1* promoter [-400 –-1 bp] (TK10036-TK10037/BY4741), and *VTC1* [+1 –+320 bp] (TK9030-TK7875/BY4741) were first amplified by PCR using the primer pair/template (genomic DNA or plasmid) as described above in parenthesis, and then fused by TK7872-TK7875 to generate a 2.5 kb fragment that was used for transformation (S3 Table). This 2.5 kb-fused fragment was then inserted at the translational initiation site (A of ATG as +1) of *VTC1* of BY4741 to generate YTK7548.

**Fig 2:**
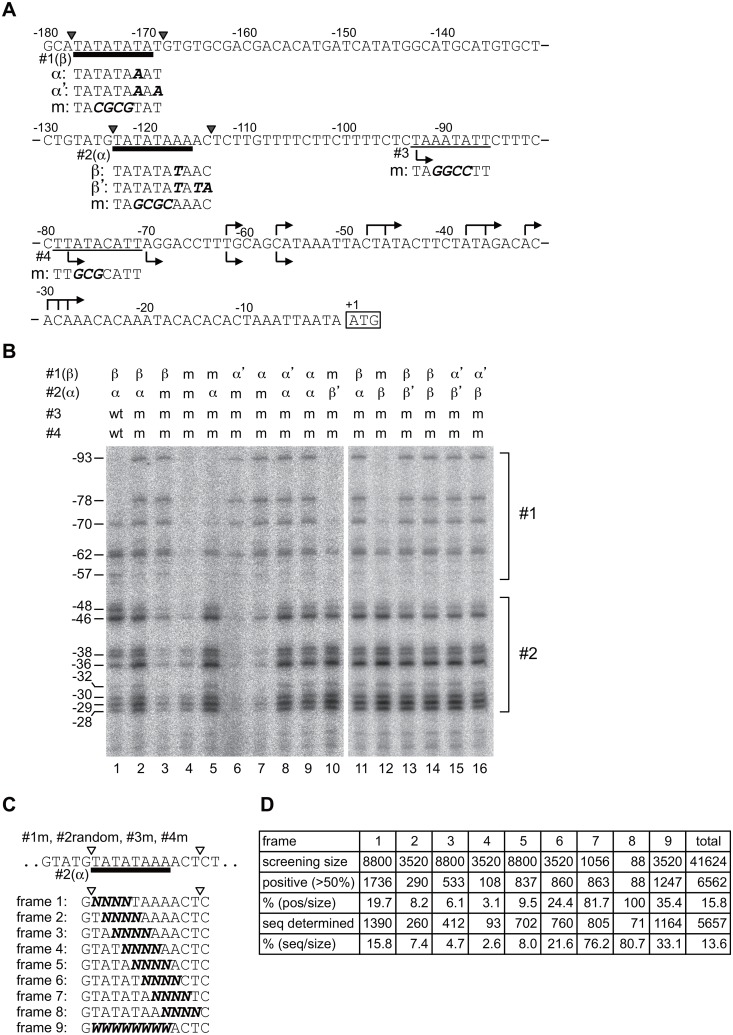
Characterization and randomization of the TATA element(s) of the *CYC1* promoter at the *VTC1* locus. **A**. Schematic diagram of the *CYC1* promoter. The positions of the four TATA or TATA-like elements are underlined as #1(β), #2(α), #3, and #4. Each TATA or TATA-like element was replaced with a specific sequence denoted as “m” to disrupt its transcriptional activity. To replace the TATAβ element (#1(β)) with the sequence(s) derived from the TATAα element (#2(α)), the region marked with two inverted black triangles was substituted for the sequence(s) denoted as “α” or “α'”. Note that “α” is identical to the TATAα element (#2(α)), whereas “α'” is identical to the sequence that had been used as the TATAα element in the original substitution study [32]. Similarly, the #2(α)-containing region marked with two inverted black triangles was substituted for the sequence(s) denoted as “β” or “β'”. The substituted nucleotides that are different from those of the wild-type are shown in “bold italic”. The arrows above or below the sequence indicate the transcription start site(s) (TSSs) that depend on the TATA element located at site #2 or #1, respectively, as shown in **B**. The site-specific distribution profiles of these TSSs are consistent with the results of a previous study [32]. The initiation codon of *CYC1* is marked with an open square along with the number +1 (A of ATG as +1). **B**. Identical function of the TATAβ and TATAα elements in the TSS selection of the *CYC1* promoter fused to the open reading frame (ORF) of *VTC1*. Total RNA (20μg) was isolated from strains containing wild-type (lane 1) or variously mutated (lanes 2–16) promoters (as indicated at the top) that had been cultured at 25°C in rich media (YPD) and subjected to primer extension analysis to determine the TSSs of the reporter constructs. The positions of the TSSs are indicated at the left (A of ATG as +1). Two sets of TSSs that are transcriptionally dependent on the TATA element(s) located at site #1 or #2 are marked with brackets at the right, and summarized graphically in **A**. **C**. Schematic diagram of the strategy used to construct nine randomized libraries (frames 1–9) that were screened in this study. The region containing the TATAα element (#2(α)), marked with two inverted open triangles, of the mutated *CYC1* promoter (#1m, #2(α), #3m, #4m as shown in lane 5 in **B**) was divided into nine frames (frames 1–9) and mutated randomly to “N” (A, C, G, T) or “W” (A, T), as indicated. **D**. Summary of the screen conducted in this study. The numbers of independent clones that were screened (screening size) and judged as active (positive (>50%)) are shown in the second and third lines, respectively. Of the active clones selected by the screen, the numbers of clones whose TATA sequences were successfully determined (seq determined) are shown in the fifth line. The percentages of “positive (>50%)” per “screening size” (pos/size) and those of “seq determined” per “screening size” (seq/size) were also calculated and are shown in the fourth and sixth lines, respectively.

To create YTK7550, the two sub-fragments containing *VTC1* [-320 –-11 bp] + *His3MX6* + *CYC1* promoter [-400 –-92 bp] (TK7872-TK10038/YTK7548) and mutated *CYC1* promoter [-116 –-1 bp] + *VTC1* [+1 –+320 bp] (TK10254-TK7875/YTK7548) were first amplified by PCR and then fused by TK2496-TK9044 to generate a 2.1 kb fragment that was inserted at the ATG site of *VTC1* of BY4741 (S3 Table). This strain (YTK7550) carried a modified *CYC1* promoter in which TATA-like sequences at sites #3 and #4 were mutated to GC-rich sequences as shown in Fig 2A.

Similarly, other strains used in Fig 2B (i.e., YTK7554, YTK7558, YTK7560, YTK7563, YTK7565, YTK7567, YTK7569, YTK7796, YTK7798, YTK7800, YTK16302, YTK16303, and YTK16304) and those used for the randomized screen (Fig 2C and 2D) were constructed by fusion PCR using the primer pair/templates described in S3 Table.

The four strains used for checking the activity of the TATTTAAA element (i.e. YTK16418, YTK16419, YTK16421, and YTK16422) were also constructed by fusion PCR, as summarized in S3 Table. Note that these strains carry the *ADE5*,*7* or *ADE6* promoter [-400 –-1 bp], instead of the *CYC1* promoter [-400 –-1 bp], to regulate the expression of *VTC1*.

All strains used in S1 Fig were derived from Y22.1, which carries a deletion of the chromosomal *TAF1* coding region and the wild-type *TAF1* gene in a *URA3*-based low-copy-number vector (pYN1) [39]. YTK2741 [40] and YTK3778 were generated from Y22.1 by replacing pYN1 with pM1169 (HA-tagged wild-type *TAF1*/pRS314) [41] and pM1746 (HA-tagged *taf1-N568Δ*/pRS314), respectively. The latter plasmid was constructed by site-directed mutagenesis of pM1169 using the primer TK176 [42]. To create YTK16455 and YTK16456, the three sub-fragments containing *VTC1* [-520 –-11 bp] (TK10267-TK7873/BY4741), *LEU2* (TK12262-TK6582/pUG73 [43]), or the *CYC1* promoter [-400 –-1 bp] + *VTC1* [+1 –+430 bp] (TK10036-TK4283/YTK7548) were first amplified by PCR, and then fused by TK10267-TK4283 to generate a 3.6 kb fragment that was used for transformation of YTK2741 and YTK3778, respectively (S3 Table). Similarly, the other four pairs of *TAF1*/*taf1-N568Δ* strains (i.e., YTK16457/YTK16458, YTK16459/YTK16460, YTK16396/YTK16397, and YTK16398/YTK16399) were generated from YTK2741/YTK3778, as summarized in S3 Table.

### Toluidine blue staining

Cells were inoculated into each well of a 96-well U-shape plate containing 100 μL of YPD media, grown overnight at 25°C without shaking, and then collected by brief centrifugation. The cell pellet was suspended in 100 μL of 80% [v/v] ethanol by shaking vigorously for 2 min. Fixed cells were collected by brief centrifugation and incubated for 10 min with 100 μL of acidic toluidine blue (TB) solution (0.1% [w/v] Toluidine Blue-O, 25% [v/v] methanol, 5% [v/v] glycerol, 5% [v/v] acetic acid, and adjusted with hydrochloric acid to pH 1.0). TB-stained cells were washed with 150 μL of DDW (deionized distilled water) three times by repeating centrifugation and resuspension. Finally, cells were suspended in 100 μL of dimethyl sulfoxide, and absorbance at 638 nm and 410 nm was measured using a Varioskan microplate reader (Thermo Scientific).

### Northern blot analysis

Northern blot analysis was performed as described previously [42]. For detection of *VTC1*, *ADE5*,*7*, *ADE6*, and *SCR1*, DNA fragments were amplified by PCR from yeast genomic DNA, purified, and ^32^P-labeled using random priming with the Klenow fragment (TOYOBO). The PCR primers used were as follows: *VTC1*/TK9030-TK9013, *ADE5*,*7*/TK8379-TK8380, *ADE6*/TK8381-TK8382, and *SCR1*/TK9507-TK10081.

### Primer extension analysis

Primer extension analysis was performed as described previously [44]. The primers used were as follows: TK9613 (+27 to +3 of *VTC1*), TK8251 (+60 to +41 of *VTC1*), TK12877 (+60 to +40 of *ADE5*,*7*), and TK12878 (+60 to +37 of *ADE6*). The cDNA products were analyzed on a 6% polyacrylamide DNA sequencing gel. Gels were exposed to imaging plates for visualization (Typhoon FLA 7000, GE Healthcare), scanning, and quantification of electrophoretic images (ImageQuant TL software version 8.1, GE Healthcare).

## Results

### A novel reporter assay system to monitor gene expression in yeast cells

Vtc1p is a subunit of the vacuolar transporter chaperone (VTC) complex that mediates polyphosphate (polyP) synthesis and transport across the membrane [45]. Our previous study showed that *VTC1* could be used as a reporter for gene expression in yeast cells through the quantification of polyP using magnetic resonance spectroscopy (MRS) and/or magnetic resonance imaging (MRI) [33]. Although these noninvasive methods are effective to measure gene expression in living cells [33, 34], they require expensive equipment and proficient skills for the analysis. Therefore, we devised a convenient method based on the same reporter gene system and the measurement of polyP amounts by staining with toluidine blue (TB) dye.

TB dye has been used extensively to detect polyP *in vivo* in various organisms [46, 47]. In addition, it is used *in vitro*, e.g., for visualization of polyP separated by polyacrylamide gel electrophoresis or for quantitative measurements of polyP synthesized enzymatically [46, 47]. As the absorption maximum of this dye is shifted to a shorter wavelength when bound to polyP (so-called metachromatic effect), the ratio of Abs_530nm_/Abs_630nm_ is used to determine the amounts of polyP *in vitro* [46, 47]. However, such quantification has not been attempted *in vivo* because the metachromatic effect could be affected by various cellular substances such as polyanions, cations, and/or proteins [46, 47].

We previously generated a set of yeast test strains carrying *VTC1* under the control of six different strength promoters (Fig 1A) and confirmed that the *VTC1* mRNA expression levels are well correlated with the amounts of polyP measured by MRS/MRI [33]. When the two test strains (#2 and #5 in Fig 1A) and the two control strains (wild-type and *Δvtc1*) were stained with TB and washed several times with water, cells exhibited strain-specific blue colors that varied depending on the amounts of polyP (Fig 1B). Although there was no clear indication for the metachromatic shift in the absorption spectra of cell suspensions, the four strains showed significantly different Abs_638nm_ (absorption maximum) values (Fig 1C). Furthermore, the Abs_638nm_ values increased proportionally at least up to 2.5 with the cell number, and the slope of each line was strain-specific (Fig 1D). By contrast, although Abs_410nm_ values increased proportionally, the slopes were the same for the four strains (Fig 1D). Collectively, these results indicate that the ratio of Abs_638nm_/Abs_410nm_ could represent a normalized value for the amount of polyP in a cell.

We inoculated the six test strains (#1–6 in Fig 1A) as well as the two control strains (wild-type and *Δvtc1*) in each well of 96-well plates in quadruplicate, and stained cells with TB after overnight incubation at 25°C (Fig 1E). The ratio of Abs_638nm_/Abs_410nm_ was determined for each well and summarized in a graph (Fig 1F, left panel). The amounts of polyP assessed by this method correlated with those of polyP determined previously by MRS [33] (Fig 1F, right panel). These results indicate that the TB-staining method could be used as a novel reporter assay system to measure gene expression in yeast cells.

### A random screen for the TATA or TATA-like elements that can direct transcription from the *CYC1* promoter

The *CYC1* promoter is one of the most extensively characterized promoters in *Saccharomyces cerevisiae* [32, 48–55] and has been shown to be a relatively rare (at least in budding yeast) poised promoter where TBP and RNA polymerase II are pre-loaded before activation [51–53, 55]. In addition, it contains several TATA or TATA-like elements, two of which, i.e. TATAβ (TATATATA located at site #1 from -177 to -170; abbreviated as β/#1) and TATAα (TATATAAA located at site #2 from -123 to -116; abbreviated as α/#2), are principally responsible for transcription from multiple transcriptional start sites (TSSs) in the upstream or downstream region, respectively [32, 48] (Fig 2A). These two TATA elements are thought to have different functions because only the upstream one is functional when the same TATA sequences are duplicated (i.e., β/#1-β/#2 or α/#1-α/#2) while both are functional when different TATA sequences are combined (i.e., β/#1-α/#2 or α/#1-β/#2) [32]. To understand this functional difference, we sought to identify any other TATA or TATA-like sequence(s) that could function as TATAβ or TATAα in the *CYC1* promoter by using a random screen based on the TB-staining method.

First, to determine whether TATAβ or TATAα have different functions also at the reporter gene (*VTC1*) locus, the wild-type or mutated *CYC1* promoter (-400 bp—+1 bp) was integrated in front of the *VTC1* coding region. The TSS(s) of the *VTC1* reporter gene were determined by primer extension analyses (Fig 2B). No differences between the function of TATAβ and TATAα at the *VTC1* locus were detected, as the TSSs of β/#1-β/#2 (lane 14) or α/#1-α/#2 (lane 9) were the same as those of β/#1-α/#2 (lane 2 and 11). As the flanking sequences were slightly modified in the original study [32], we reconstructed the strains by exploiting the same sequences as the original ones (designated here as TATAβ' and TATAα'). The results showed that the TSSs of β/#1-β'/#2 (lane 13) or α'/#1-α/#2 (lane 8) were the same as those of β/#1-α/#2 (lane 2 and 11), α'/#1-β'/#2 (lane 15), and α'/#1-β/#2 (lane 16), leading to the same conclusion that all four TATA elements (i.e., β, α, β', α') have an identical function at the *VTC1* locus, which is different from their function at the original *CYC1* locus. A previous study [32] showed that, when the *CYC1* promoter contains a single TATA at either site #1 or #2, position-specific and sequence-independent conventional TATA function is observed at the *CYC1* locus. This function was confirmed at the *VTC1* locus, as the TSSs of β/#1-mut/#2 (lane 3) or mut/#1-α/#2 (lane 5) were the same as those of [α or α']/#1-mut/#2 (lane 6, 7) and mut/#1-[β or β']/#2 (lane 10, 12), respectively. Therefore, we could recapitulate the sequence-independent function but not the sequence-dependent function of these two TATA elements at the *VTC1* locus. Furthermore, the functions of TATAβ and TATAα were found to be TFIID-dependent at the *VTC1* locus (S1 Fig), which is contrary to the previous assumption that the *CYC1* promoter is TFIID-independent [25, 42, 55, 56]. In fact, we confirmed that the endogenous *CYC1* promoter is TFIID-independent even in the strains used in this study (data not shown). Thus, the chromosomal context and/or promoter structure outside the substituted region may alter the selectivity for the core transcriptional machineries (e.g., TFIID vs. SAGA), thereby generating the aforementioned discrepancies between the previous [32] and current studies. In any case, information on preferred TATA or TATA-like sequence(s) in a TFIID-dependent promoter is limited [20, 35]. Therefore, despite its deviation from the original intention, we performed a random screen to identify functional core promoter element(s) that could substitute for TATAα in the *CYC1* promoter (mut/#1-α/#2-mut/#3-mut/#4) bearing nearly identical transcriptional activity to the wild-type (β/#1-α/#2-wt/#3-wt/#4 in Fig 2B and S1 Fig).

**Fig 3:**
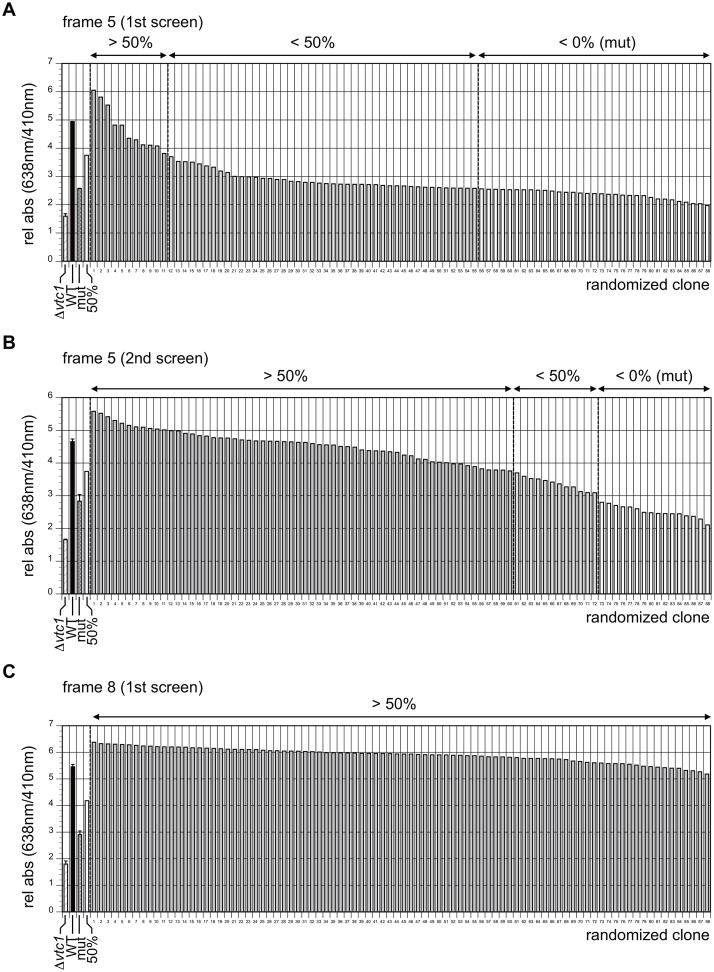
Active clones isolated by the screen when frame 5 or 8 was randomized. **A**. One part (corresponding to 88 independent clones) of the first screen for frame 5 (8,800 independent clones in total) conducted in a 96-well microplate format is shown as an example to demonstrate the profiles of the three types of clones (>50%, <50%, <0%) that can be obtained in the screen. Promoter activities (i.e., relative absorption at 638 nm/410 nm) of the wild-type strain (#1m, #2α, #3m, #4m) and the mutated strain (#1m, #2m, #3m, #4m) are set as 100% (second bar) and 0% (third bar), respectively. The clones showing >50% activity (more than the value of the fourth bar = 50%) were judged as active and pooled for the second screen as shown in **B**. The clones showing <50% activity (including those less than 0%) were judged as inactive and not used for further analyses. All clones are aligned in order of promoter activities. Note that the promoter activity of the mutated strain (0%) is not completely inactive, as it is stronger than that of the *Δvtc1* strain (first bar). Consistent with this, a significant number of clones showed promoter activities <0%. **B**. A part of the second screen for frame 5 is shown as an example to demonstrate the profiles of the three types of clones (>50%, <50%, <0%). The population size of the >50% clones becomes larger when compared with that of the first screen as shown in **A**, indicating that the active clones were enriched significantly via multiple screening. **C**. First screen for frame 8 (88 clones in total). The results indicate that all clones were active, contrary to those for frame 5 as shown in **A**.

The TATATAAAACT (TATAα is underlined) sequence was divided into nine frames (frame 1–9), and four (N = A/C/G/T) or eight (W = A/T) nucleotides of each frame were randomized by PCR as shown in Fig 2C. The screen was conducted in a 96 multi-well plate format, each of which contained 88 (or less) distinctly randomized strains as well as the three control strains (in duplicate), i.e., wild-type (WT, positive control), a TATA mutant (TA*GCGC*AAACT, negative control), and *Δvtc1*. The TB-staining method is quantitative enough to compare the strains processed in the same plate (i.e., under the same staining and washing conditions). However, this method could not provide an absolute value for the activity, and the results obtained at different experimental dates were difficult to compare. Therefore, the activity of each clone was evaluated by comparing it with those of the control strains processed in the same plate at the same time. In the screen, the clones showing more than the average (50%) activity of the WT and TATA mutant were judged as potentially active (Fig 3A), and those that passed through the selection twice (Fig 3B) were stored as active clones and subjected to the sequence analysis. Active clones were significantly enriched through such multiple selection processes (compare Fig 3A and 3B). The results of the screen are summarized in Fig 2D. In total, 6,562 active clones (15.8%) were isolated from 41,624 randomized clones. Among them, 5,657 clones (13.6%) were successfully sequenced and tentatively classified into the nine groups, as described below.

### Requirements for the 7^th^ or 8^th^ position of the TATA element in the *CYC1* promoter

**Fig 4:**
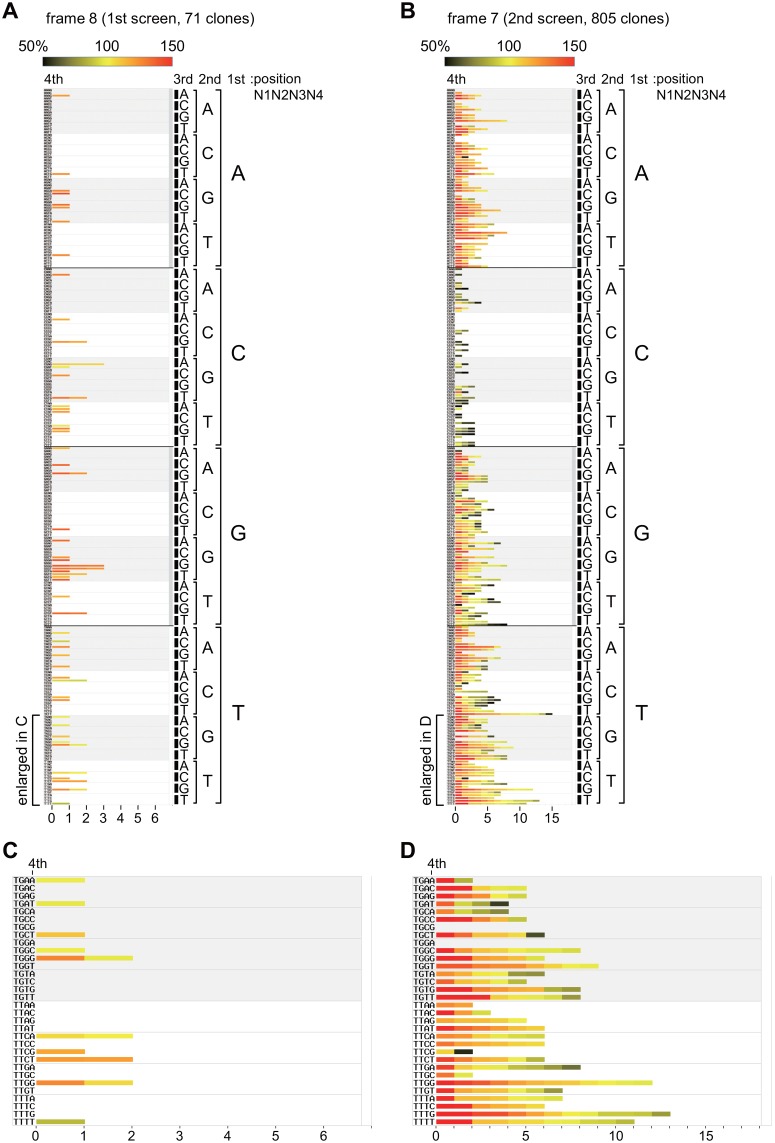
Graphical summary of active clones isolated by the screen for frames 7 and 8. **A**. Characterized properties (activity, sequence, and isolation frequency) of the active and successfully sequenced 71 clones that were isolated by the first screen for frame 8 (S6 Table) are graphically summarized in a heat map. Each clone is represented by a small square painted with an appropriate color according to its promoter activity, as indicated at the top (WT = 100%, mut = 0%), and arranged to a specific site of this heat map according to its sequence of the randomized region (TATATAA*N* ^*1st*^ *N* ^*2nd*^ *N* ^*3rd*^ *N* ^*4th*^). Note that the squares are aligned in order of promoter activities from left to right (i.e., each bar has a red to gray color as shown more evidently in **B**, **D**). The numerals along the horizontal axis indicate the number of clones isolated by the screen. The area enlarged in **C** for readability of the sequence is marked with a bracket. **B**. Characterized properties (activity, sequence, isolation frequency) of active and successfully sequenced 805 clones that were isolated by the second screen for frame 7 (S5 Table) are summarized in a heat map. Small squares representing each clone with an appropriate color are arranged in the same way as described in **A**. The area enlarged in **D** is also marked with a bracket. **C**. The area bracketed in **A** was enlarged. **D**. The area bracketed in **B** was enlarged.

A previous study revealed the consensus sequence TATAWAWR (W = A/T, R = A/G) for the TATA element [19]. However, the clones obtained in the screen for frame 8 were all active (Fig 3C), indicating that the requirements for the 8^th^ position (i.e., “R” (R = A/G) in the consensus sequence) are greatly alleviated in the *CYC1* promoter. To confirm this, we sequenced 71 active clones and found that no sequence bias was generated during the construction process (Fig 4A, S6 Table). Thus, we conclude that the length of the TATA element in the *CYC1* promoter is 7 bp, unlike that of the consensus TATA (8 bp). A similar conclusion was reached in a previous study in which 23 TATAα mutants were assayed *in vivo* and *in vitro* [54].

In the screen for frame 7, 805 (76.2%) active and successfully sequenced clones were obtained from 1,056 randomized clones (Fig 2D). The isolation frequency of active clones was significantly higher than that expected (approx. 50%) from “W” (W = A/T) in the consensus TATA, suggesting that the requirements for the 7^th^ position are also alleviated in the *CYC1* promoter. A heat map for these 805 clones clearly showed that the 7^th^ position in this promoter prefers “D” (D = A/G/T) instead of “W” (W = A/T) in the consensus TATA (Fig 4B, S5 Table). Our study revealed for the first time that the *CYC1* TATA disfavors “C” at the 7^th^ position, although previous findings showed that the *CYC1* TATA is active when it has “G” at the 7^th^ position [54].

### Classification of active TATA or TATA-like sequences obtained by the screen

**Fig 5:**
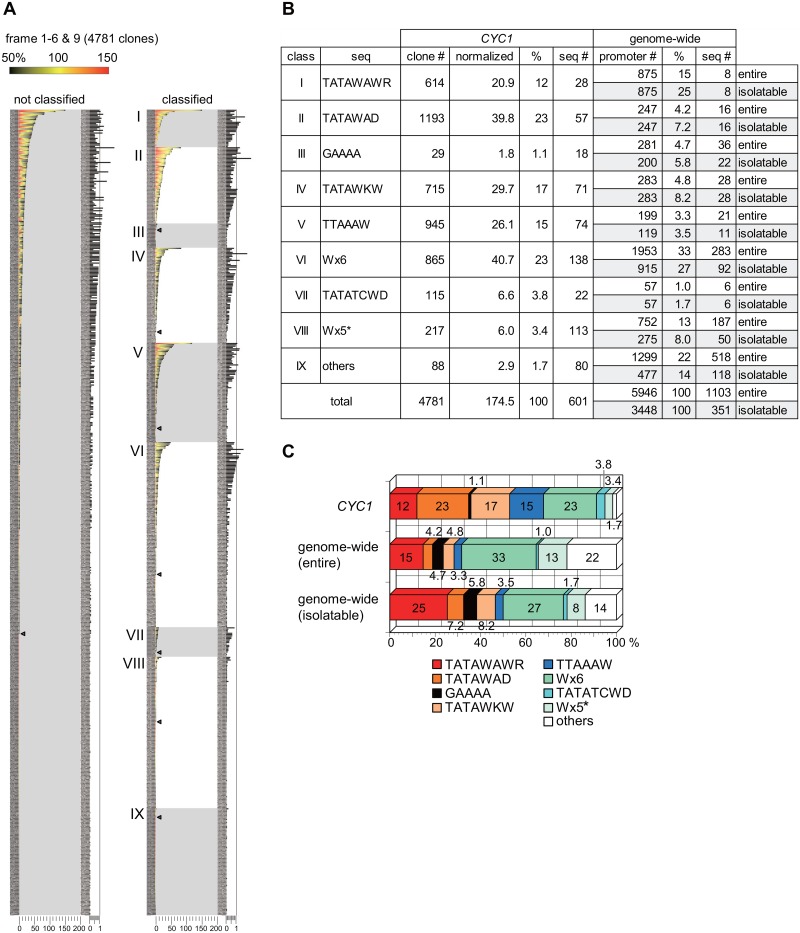
Classification of active clones and comparison with the PIC assembly sites on the genome. **A**. Graphical summary of all active clones isolated by the screen for frames 1–6 and 9 (S4 Table). In the left panel, the clones carrying the same sequences (as indicated at the left) were combined, regardless of which frame they were derived from, and aligned in order of the total clone number. The color code for each clone is the same as that described in Fig 4A. To compare the isolation frequency among the sequences, normalization is required since the chance to be isolated as an active clone is proportional to the numbers of each clone existing in each library. For this purpose, the expected number of a given clone carrying a specific sequence generated in each library was calculated and summed up across all frames (i.e., frames 1–6 and 9). The actually isolated clone number carrying each sequence was divided by each expected clone number to determine the normalized isolation frequency (S7 Table). This normalized value for each sequence is shown at the right side of the left panel with a black bar. The value of 1 (as indicated with a vertical thin line) represents 100% recovery; that is, the clones carrying a given sequence that exist in the library were all recovered as active clones by the screen. Note that some clones/sequences exceed the value of 1, indicating that there may be a certain bias to generate some specific clones/sequences more frequently than others during the randomization process. The numerals along the horizontal axis indicate the clone number (left, 0–200) or the normalized isolation frequency (right, 0–1). In the right panel, the clones presented in the left panel were classified into nine groups as summarized in **B** and aligned in order of the total clone number in each class. This panel was enlarged in S2–S4 Figs for readability of each sequence. In both panels, the boundary between the clones isolated more than twice and those isolated only once is marked with a closed triangle, highlighting the reproducibility of each clone in the screen. **B**. Summarized table for classification of the clones/sequences (9 bp) isolated by the screen and their comparison with those of the PIC assembly sites (8 bp) on the genome [27]. The number of the isolated clones (indicated as “clone #”), the sum (integral value) of the normalized isolation frequency (normalized value in S7 Table) for each sequence in each class (indicated as “normalized”), the percentage of each summed “normalized” value in total (indicated as “%”), and the number of the distinct sequences belonging to each class (indicated as “seq #”) are summarized in the left-middle four columns denoted as “*CYC1*” at the top. The “entire” set of sequences (8 bp) identified previously as the PIC assembly sites [27] were classified into two groups that could be isolated by our screen (“isolatable”) or not (“not isolatable”). The “entire” or “isolatable” sequences were further classified into the same nine groups as those isolated by the screen. The number of the sequences (indicated as “seq #”), the number of the genes containing such sequences in the promoter region (indicated as “promoter #”), and the percentage of the latter belonging to each class in total (indicated as “%”) are summarized in the right three columns denoted as “genome-wide” at the top. Abbreviations are as follows: W = A or T; R = A or G; D = A or G or T; K = G or T; Wx6 = WWWWWW; Wx5* = WWWWW except the clones/sequences included in class VII. **C**. The percentages of the “normalized” value (*CYC1*) and those of the “promoter #” (genome-wide) were presented graphically.

In the screen for frames 1–6 and 9, 4,781 (11.8%) active and successfully sequenced clones were obtained from 40,480 randomized clones (Fig 2D). These clones comprised 601 different sequences (Fig 5B) and were summarized in a heat map with the normalized value (left panel in Fig 5A, S4 Table). Here, the normalized value for each sequence was calculated by taking into account which frame(s) could generate such a sequence, since the expected isolation frequency should vary depending on the population size of each sequence in the library (see the details in the legend for Fig 5A, S7 Table).

To further characterize 601 potentially active sequences, we sought to classify 4,781 clones into several groups. As described above, TATAWAWR (consensus TATA) and TATAWAD were both shown to be active in the *CYC1* promoter. Thus, 28 sequences (614 clones) that matched TATAWAWR were first extracted, followed by 57 sequences (1,193 clones) that matched TATAWAD successively (class I and II in Fig 5A and 5B, S2A and S2B Fig). It was previously shown that the GA element (GAAAA) is a novel type of core promoter element that does not co-occur with the TATA element [57]. Thus, we next extracted 18 sequences (29 clones) that match GAAAA (class III in Fig 5A and 5B and Nx9 in S2C Fig) from the rest (2,974 clones). Our classification analyses searched for the sequence(s) within the region from -123 to -115 (9 bp: originally TATATAAAA) that included “N” (frame 1–6) or “W” (frame 9) (Fig 2C; TATAα is underlined). As the 5′-upstream nucleotide adjacent to this region was “G” (Fig 2C), we also searched for the sequences that matched GAAAA within the 1 bp-extended region (10 bp: GTATATAAAA) and extracted 25 sequences (54 clones) (G/Nx9 in S2C Fig). The activities of these sequences were considerably weaker than those of TATAWAWR or TATAWAD (compare the heat maps in S2 Fig). In addition, the reproducible (i.e., isolated more than twice) clone numbers for these sequences were significantly smaller than those for the latter two TATA-containing sequences, suggesting that the GA element may not be functional in the *CYC1* promoter.

Next, we searched for the TATA-containing sequence(s) other than TATAWAWR and TATAWAD by using TATAN (N = A/G/C/T) as an initial probe and found that the sequences containing TATAW (W = A/T) were considerably more active than those containing TATAS (S = G/C) by examining the color profile and clone number in the heat map (data not shown). Furthermore, TATAWD (D = A/G/T) was found to be a better sequence motif than TATAW, since the sequences containing TATAWD were significantly more active than those containing TATAWC (data not shown), although the latter matched another less active sequence motif, i.e., TATATCWD (class VII), as described later. Based on the same reason, TATAWDW was a better motif than TATAWD when the heat maps of TATAWDW (W = A/T) and TATAWDS (S = G/C) were compared (data not shown). Finally, TATAWKW (K = G/T) was chosen as a sequence motif representing class IV, as TATAWAW was already included in TATAWAD (class II), and further extension was not informative (i.e., no differences were found for TATAWKWA, -C, -G or -T) (data not shown). We extracted 71 sequences (715 clones) that matched this motif (class IV in Fig 5A and 5B and S3A Fig) and found that class IV was less active than class I and II (S2A, S2B and S3A Figs).

After exclusion of the sequences belonging to class I–IV from the total (4,781 clones), the top five most frequently isolated sequences among the remaining clones (2,230 clones) contained TTAAA (data not shown). Thus, we next searched for TTAAA-containing sequence(s) and found 945 clones containing TTAAAW (W = A/T), while no clones containing TTAAAS (S = G/C) were identified (data not shown). In addition, most of them (751 clones) had TTAAAW at the 3′-end (i.e., further extension was not informative), indicating that TTAAAW is another sequence motif. In fact, 74 sequences (945 clones) that matched this motif (class V in Fig 5A and 5B, S3B Fig) were extracted, and class V was less active than class I and II, but more active than class IV (S2A, S2B, S3A and S3B Figs).

No significant sequence features were detected in the remaining clones (1,285 clones) except that they were all W (= A/T)-rich. When 138 sequences (865 clones) that matched Wx6 (class VI) and then 135 sequences (332 clones) that matched Wx5 (class VII+VIII as described below) were extracted successively, we noticed that the latter class included a significant number of TATATC-containing sequences (data not shown). As most of these sequences could be represented as TATATCWD (D = A/G/T), the latter Wx5 class was divided into two subclasses, i.e., TATATCWD (22 sequences/115 clones) and Wx5* (= Wx5 other than TATATCWD; 113 sequences/217 clones) (class VII and VIII, respectively, in Fig 5A and 5B, S4B and S4C Fig). The remaining 80 sequences (88 clones) were classified as “others” (class IX in Fig 5A and 5B and S4D Fig). It is likely that class IX is not functional, since most of the clones (73 clones) were isolated only once in the screen, presumably because of experimental noise. Collectively, we conclude that active sequences/clones could be classified into seven groups, namely, class I (TATAWAWR), II (TATAWAD), IV (TATAWKW), V (TTAAAW), VI (Wx6), VII (TATATCWD), and VIII (Wx5*) in the *CYC1* promoter. However, each class may include a certain number of false-positive clones (e.g., those isolated only once) since the TB-staining method is semi-quantitative and could not yield an absolute value for the activity. Consistent with this, even the same sequences (including the wild-type “TATATAAAA” sequence that was isolated most frequently in the screen) exhibited apparently varied activities (see color profiles in Fig 5A and S2–S4 Figs).

### Comparison of active sequences isolated by the screen with those identified as the PIC assembly sites throughout the genome

Recently, Rhee and Pugh determined the precise positions for a number of PICs (6,045) on the yeast genome by using a ChIP-exo method [27]. They found that nearly all of the PICs were assembled over the region including 8 bp DNA (judged from the position of TFIIB) that showed two or less mismatches to the consensus TATAWAWR even in the TATA-less promoters. Mismatched sequences are designated formally as TATA-like elements [27].

Here, we sought to compare the sequences isolated by the screen with the TATA or TATA-like elements identified previously by the ChIP-exo method. Given that the entire set of these genome-wide identified elements were not included in our library, we set up two categories for the comparison, i.e., “entire” and “isolatable” (Fig 5B and 5C). The latter set included only the sequences that could be isolated by the screen. First, we classified the “entire” set (5,946 sites) of the PIC assembly sites (http://downloads.yeastgenome.org/published_datasets/Rhee_2012_PMID_22258509/track_files/) into the same nine groups (class I–IX) and found that the population sizes of classes II (TATAWAD, 4.2%), IV (TATAWKW, 4.8%), and V (TTAAAW, 3.3%) were significantly smaller than those of classes I (TATAWAWR, 15%), VI (Wx6, 33%), and VIII (Wx5*, 13%) (Fig 5B and 5C). A similar tendency was observed for the “isolatable” set (3,448 sites) when it was classified into the same nine groups (Fig 5B and 5C). In addition, a significant number of sites were classified into class IX (others, 22%[entire] or 14%[isolatable]), contrary to the screen, in which the number of active clones classified in this class was almost zero, indicating that class IX sequences are disfavored specifically by the *CYC1* promoter. By contrast, the clones/sequences in classes II, IV, and V appeared to be significantly active in the *CYC1* promoter (Fig 5A) and thereby occupied the major fraction of the isolated clones in the screen (Fig 5B and 5C). Furthermore, the population size of the TATATCWD sequences (class VII) in the Wx5 sequences (class VII+VIII) was more than 50% (normalized value) for the screen but only 7% (= 57/809, entire) or 17% (= 57/332, isolatable) for the genome-wide analysis (Fig 5B and 5C), indicating that the TATATCWD sequences are favored specifically by the *CYC1* promoter. Altogether, these results imply that the preferred sequence(s) vary among the genes, presumably depending on the promoter structure. Notably, the population size of class III (GAAAA) was smaller in both the screen (1.1%) and the genome-wide analysis (4.7%[entire] or 5.8%[isolatable]) than the size expected based on the previous notion that the GA element is found frequently in the core promoter region (37.3%) [57]. Thus, we speculate that this element must have a different function other than serving as the PIC assembly site.

There are two types of core promoters in budding yeast that are Taf1p-enriched or Taf1p-depleted [27]. The population size of class I (consensus TATAWAWR) was significantly different for these two types of core promoters; it was much smaller in the former (9.6%[entire] or 17%[isolatable]) than in the latter (37%[entire] or 53%[isolatable]) (S5 Fig) [27]. The corresponding value (12%) obtained in the screen was also small, suggesting that the *CYC1* promoter is closer to the Taf1p-enriched type than to the Taf1p-depleted type. The significance of the difference between the screen and genome-wide “isolatable” data for each class was evaluated by using the Z test. The calculated p-values are summarized in S5A Fig. This is consistent with the fact that the *CYC1* promoter showed Taf1p-dependency when it was integrated at the *VTC1* locus (S1 Fig).

The normalized isolation frequencies (modified slightly from those in Fig 5A as described in the legend for S6 Fig) for several class I or II sequences were compared individually with the number of PIC assembly sites containing such sequences in the Taf1p-enriched and/or Taf1p-depleted core promoters (S6 Fig, note that all of these sequences are “isolatable”). As expected, the class II sequences were favored by the *CYC1* and Taf1p-enriched core promoters compared to the Taf1p-depleted core promoters. Furthermore, the normalized isolation frequency for each sequence in the screen did not correlate with its utilization frequency as the PIC assembly site on the genome. These observations suggest that the preferred sequence(s) as the core promoter element may vary among the genes, presumably depending on the promoter structure even within the Taf1p-enriched type.

### TATTTAAA is a functional core promoter element of endogenous genes

**Fig 6:**
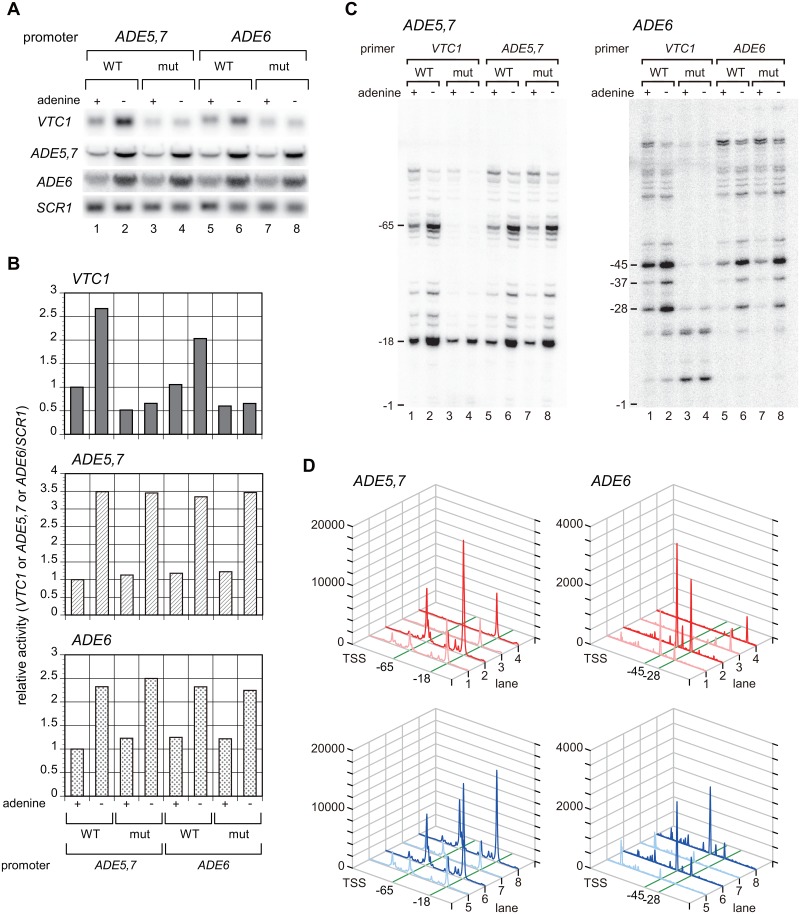
The TATTTAAA sequence functions as a core promoter element in the *ADE5*,*7* and *ADE6* promoters. **A**. Northern blot analyses to examine the expression of the reporter (*VTC1*) or control genes (*ADE5*,*7*, *ADE6*, and *SCR1*) in strains carrying the reporter driven by the wild-type (WT: lane 1, 2) or mutated (mut: lane 3, 4) *ADE5*,*7* promoter, or similarly by the wt (lane 5, 6) or mut (lane 7, 8) *ADE6* promoter, as indicated at the top. Total RNA (20 μg) was isolated from these strains cultured at 30°C in synthetic complete (SC) media with (+) or without (-) adenine, blotted onto the membrane, and hybridized with the gene-specific probes indicated at the left. **B**. The raw data shown in **A** were quantified and presented graphically. Values for each transcript (*VTC1*, *ADE5*,*7*, *ADE6*) were normalized by *SCR1* (pol III transcript) and presented as relative to those of the strain carrying the reporter driven by the wt *ADE5*,*7* promoter and cultured in the presence of adenine (i.e., the values at the most left side are set as 1). **C**. Primer extension analyses to examine the transcriptional start site(s) (TSSs) of the reporter (*VTC1*) and endogenous genes (*ADE5*,*7*, or *ADE6*) in the same strains as described in **A**. Total RNA (20 μg) was subjected to the primer extension analysis using the specific primers for *VTC1*, *ADE5*,*7*, or *ADE6* as indicated at the top. The positions of major TSSs relative to the A (+1) of the start codon ATG are indicated at the left of each panel. Note that the primers for *VTC1* or endogenous genes (*ADE5*,*7* or *ADE6*) were designed to generate the same size of cDNA fragments when mRNAs were transcribed from the same TSSs. The TSS profiles along with the promoter sequences (wt, mut) of *ADE5*,*7* and *ADE6* are schematically shown in S7 Fig **D**. Each lane of the electrophenograms shown in **C** was scanned, quantified by densitometry (ImageQuant TL software version 8.1, GE Healthcare), and presented graphically. The results for the reporter (*VTC1*, lanes 1–4) are shown in the upper two panels, while those for the endogenous genes (*ADE5*,*7* or *ADE6*, lanes 5–8) are shown in the lower two panels.

Class V represents a unique sequence motif that is significantly active, even though it does not contain any “TATA” sequences. The most frequently isolated and the most active sequence in this class was “TATTTAAAA” (9 bp) (S3B Fig). To examine whether this sequence serves as a TATA-like element even in the endogenous genes, we searched for “TATTTAAA” or “ATTTAAAA” among the PIC assembly sites (8 bp) on the genome [27] and found that only the former functions at the 35 distinct promoters (data not shown). Among them, two promoters (i.e., *ADE5*,*7* and *ADE6*) were selected to determine whether the sequence “TATTTAAA” was functional (S7 Fig). Northern blot analyses showed that the reporter gene (*VTC1*) fused to these promoters could be activated at similar levels to the endogenous genes under the adenine-starved condition (Fig 6A and 6B) [58]. The “CGCCCGGG” substitution of this element (S7 Fig) significantly impaired the basal and activated expression of these two promoters (Fig 6A and 6B). Primer extension analyses also showed that this element plays a pivotal role in supporting the normal levels of basal and activated expression from the accurate TSSs in these two promoters (Fig 6C and 6D). Collectively, these results indicate that at least some of the sequences isolated by the screen are utilized as the core promoter element(s) for the endogenous genes.

## Discussion

### A novel reporter assay system using toluidine blue (TB) dye

In the present study, we developed a novel reporter assay system to monitor gene expression in yeast cells. This method is semi-quantitative but simple, convenient, and inexpensive, since it uses only TB dye for visualization. This enables rough estimation of promoter activities without equipment, simply by judging the color with the naked eye (Fig 1B and 1E). Several reporter assay systems using β-galactosidase (β-gal), luciferase (Luc), or GFP (or its derivatives) are well established in yeast [59–64]. These methods require expensive measuring equipment such as fluorometers, luminometers or flow cytometers. The β-gal activity can be measured by spectrophotometer when colorimetric substrates (e.g., ONPG or X-gal) are used in the assay. However, the procedures for cell disruption/permeabilization and measurement of enzymatic activities within the linear range are time-consuming and laborious. Reagents for reducing such labors are commercially available; however, they are expensive compared to those needed for the TB assay. Another distinctive feature of the present method is that the target molecule for the measurement is not the protein itself (i.e., unlike β-gal, Luc, or GFP), but rather a biochemical product (i.e., polyP) accumulated endogenously in the cell by the action of the reporter gene (i.e., *VTC1*). Thus, this method has advantages over other methods in that it does not require any exogenous substrates or intense labor efforts to adjust the assay within the linear range (unlike β-gal or Luc), or expensive equipment (unlike Luc or GFP) for the measurement. However, this method is not adequate for following the dynamic behavior of target promoters because polyP is stable after the shutoff of *VTC1* expression [33], although it is useful to measure promoter activity in a steady state as shown in this study.

Recently, massively parallel reporter assay (MPRA) systems have been developed owing to the advancement of next-generation sequencing technologies [65–70]. In these assay systems, promoter activities can be measured directly by sequencing cDNAs tagged individually with a specific barcode for identification. Such tagging system enables each promoter activity to be measured even in the mixed population, thereby greatly increasing the efficiency of the method to identify active promoter elements. Despite these advances, efforts to reveal the structure and function of the core promoter elements remain limited [65], as most studies have been focused on the functional properties of the UAS/enhancers [66–70]. Recently, a genome-wide approach using a STARR-seq (self-transcribing active regulatory region sequencing) method [70] demonstrated that there are three types of enhancers in *Drosophila*, namely, those that can specifically activate a “housekeeping-type” core promoter containing the TCT motif (hkCP enhancer), those that activate a “developmental-type” core promoter containing TATA, Inr, MTE, and DPE (dCP enhancer), or those that activate both (shared enhancer) [12]. This has enabled the identification of core promoter element(s) that can be activated by a specific type of UAS/enhancer in a more systematic manner. Although the MRPA and/or STARR-seq methods would be ideal for such a purpose, they are unaffordable for most researchers. In this regard, the TB method still has competitive merits, especially in budding yeast, owing to its cost-effective and high-throughput features that may be enough for the analysis of organisms carrying a compact genome equipped with fewer regulatory *cis*-elements.

### TATAWAD is a functional TATA element motif for the *CYC1* promoter

A previous study demonstrated that the consensus TATA sequence is TATAWAWR (8 bp), in which the 8^th^ position is confined to “R” (R = A/G) [19]. By contrast, we showed that any base could function almost equally at the 8^th^ position (Figs 3C and 4A), indicating that the TATA element is 7 bp at least in the *CYC1* promoter. This assumption is consistent with a previous study showing that TATATAAN (N = A/G/C/T) are all functional at the TATAα site in the *CYC1* promoter *in vitro* and *in vivo* [54]. We also showed that the base at the 7^th^ position is critical for function but is not confined to “W” (W = A/T), as expected from the consensus TATA sequence. This position allowed “G”, but not “C”, to be fully active (Fig 4B), implying that the 7^th^ position should be assigned to “D” (D = A/G/T) at least in this promoter. Therefore, we conclude that the TATA sequence preferred by the *CYC1* promoter should be represented as “TATAWAD” (7 bp) rather than the consensus “TATAWAWR” (8 bp). To the best of our knowledge, this is the first demonstration of the “functional consensus TATA sequence” determined experimentally for one particular promoter, i.e., by using a functional screen to select for a large number of transcriptionally active clones from a randomized library. Of note, a similar randomized screen was conducted previously for the T_R_ element of the *HIS3* promoter [35]. However, functional consensus TATA sequence(s) could not be determined for this promoter, probably because a broader region containing the TATA element (16 bp) was replaced at once with the randomized sequence(s), contrary to our strategy in which the TATA element was divided into multiple small segments (frames 1–8) for randomization (Fig 2C).

### Promoter specificity of the TATA or TATA-like sequences that were isolated by the screen as the TATAα element of the *CYC1* promoter

The populations containing the sequences that matched either TATAWAWR (class I) or TATAWAD (class II) showed similar sizes in the screen for the active *CYC1*-TATAα element (class I+II = 35%) and the “isolatable” set of the PIC assembly sites (class I+II = 33%) (Fig 5C). However, the ratio of class II to I of the former (class II/I = 1.9) was significantly higher than that of the latter (class II/I = 0.28), indicating that TATAWAD is a less frequently utilized “functional TATA element motif” than TATAWAWR on the genome. Namely, the requirements for the 7^th^ and/or 8^th^ position(s) of the TATA element could be alleviated only for a limited number of genes. In this regard, it should be noted that the well-characterized TATA element of *HIS3* (T_R_: TATAAAGT) [29] matched TATAWAD but not TATAWAWR. Furthermore, the T_R_ element remains active when “G” at the 7^th^ position is mutated to “A” or “T”, but not when it is changed to “C” [29, 71]. These observations suggest that both the *HIS3*-T_R_ and *CYC1*-TATAα elements might belong to the same TATA element subfamily for which TATAWAD could represent a “functional consensus TATA element motif”. Intriguingly, a detailed structural study of the TBP-TATA complex demonstrated that “C” or “G” at the 7^th^ position could induce a Hoogsteen-type base pair instead of a conventional Watson-Crick-type base pair upon TBP binding [72]. Thus, TATAWAD might be a novel type of core promoter element carrying a different function from that of the consensus TATAWAWR.

This study also showed that the function of the *CYC1*-TATAα element became Taf1p-dependent when it was integrated at the *VTC1* locus (S1 Fig). Contrary to these findings, the function of the *HIS3*-T_R_ element is known to be Taf1p-independent [73], arguing against the possibility that TATAWAD is a functional TATA element motif for Taf1p-dependent promoters. However, the region (TATATAAAGT) containing the *HIS3*-T_R_ element (underlined sequence) of the *HIS3* promoter matches both TATAWAD (TATATAAAGT) and TATAWAWR (TATATAAAGT). The Taf1p-dependency of the *HIS3*-T_R_ element was examined within the context of the original promoter [73], while mutational studies of the *HIS3*-T_R_ element were conducted for the shorter TATA segment containing TATAAAGT (TATAWAD-matched sequence) but not TATATAAA (TATAWAWR-matched sequence) [29, 71]. Therefore, it remains possible that the function of the *HIS3*-T_R_ element itself (TATAAAGT) is Taf1p-dependent, and, if so, TATAWAD might be a functional TATA element motif for the Taf1p-dependent promoters. This assumption is consistent with the observation that the utilization ratio of TATAWAD to TATAWAWR at the PIC assembly site is significantly higher in the Taf1p-enriched promoters than in the Taf1p-depleted promoters (S5 Fig). Further analyses are needed to test this possibility more directly.

Another remarkable observation of the present study is that TTAAAW-containing sequences (class V) could function as active core promoter elements in the *CYC1* promoter (Fig 5A, S2–S4 Figs). This motif also appeared to be gene-specific by comparison with the genome-wide data (Fig 5C). We paid special attention to the sequence “TATTTAAAA” (1 bp mismatched from the wild-type “TATATAAAA”) because it was the most frequently isolated, not only in class V but also across the entire classes (class I–IX) except the wild-type sequence itself (Fig 5A, S2–S4 Figs). Furthermore, this sequence was one of the most active sequences among the 23 specifically mutated *CYC1*-TATAα elements [54]. Notably, a similar but non-class V variant, “TATTTAGT” (2 bp mismatch) of the *HIS3*-T_R_ element, was shown to be as active as the wild-type [29], and another variant, “TATTTAAT” (termed R2; 3 bp mismatch), was isolated as one of the most active *HIS3*-T_R_ elements by a randomized screen [35]. These observations suggest that “TATTTARW” (R = A/G, W = A/T) provides a novel core promoter element motif that is shared by the *HIS3* (TATTTAGT, TATTTAAT) and *CYC1* (TATTTAAA*A*) promoters. However, as the sequences containing this motif were isolated less frequently by our screen (i.e., TATTTAATA/class VI [10 clones/normalized value 0.73], TTATTTAAA/class VI [8 clones/normalized value 0.58], ATATTTAAA/class VI [4 clones/normalized value 0.29] and TATTTAGAA/class VI [2 clones/normalized value 0.15] other than TATTTAAAA/class V [118 clones/normalized value 1.07]), and the isolated clones appeared to be rather inactive except the “TATTTAAAA” sequence itself (S4 and S7 Tables), we assume that “TTAAAW” (class V) is a more appropriate core promoter element motif than others, such as “TATTTARW”, at least for the *CYC1* promoter. Notably, three class V sequences, namely, “CACCGCTATTTAAATCCC” (termed R15), “CTACTACTATTAAAACCCA” (termed R33), and “TTAAAAGCGTCCCATTTC” (termed R51), were isolated as *HIS3*-T_R_ derivatives by a randomized screen, although their activities were very weak [35], supporting the aforementioned view that class V may represent a gene-specific core promoter element motif.

We found that the “TATTTAAA” (8 bp) sequence was utilized as the PIC assembly site in 35 endogenous genes [27] and demonstrated that this sequence could function as a *bona fide* core promoter element at least in the *ADE6* and *ADE5*,*7* promoters (Fig 6). Of note, when the frame containing this 8 bp sequence was extended downstream by 1 bp, the “TATTTAAAC” and “TATTTAAAT” sequences resided in the *ADE5*,*7* and *ADE6* promoters, respectively (S7 Fig). The PIC was also assembled on the same “TATTTAAA” (8 bp) sequence in the *ADE4* promoter [27], and a similar 1 bp extension revealed the “TATTTAAAG” sequence in this promoter. These observations suggest that these three *ADE* genes (i.e., *ADE4*, *ADE5*,*7*, and *ADE6*), which are involved in the early steps of the purine *de novo* synthesis pathway [74], might be regulated coordinately in a “TATTTAAA” motif-dependent manner rather than in a “TTAAAW” motif-dependent manner. Our screen could isolate “TATTTAAAA” but not “TATTTAAA[C/G/T]”, as the latter was not included in the library, implying that the 9^th^ position of the “TATTTAAAA” sequence may not be important for *CYC1* promoter activity, similar to the *ADE* promoters. However, “TATTTAAA”-containing sequence(s) (i.e., TTATTTAAA/class VI [8 clones/ normalized value 0.58] and ATATTTAAA/class VI [4 clones/normalized value 0.29]) were isolated less frequently and showed weaker activities than “TATTTAAAA” as described above (S4 and S7 Tables), indicating that “TATTTAAAA” (9 bp) is a more appropriate functional core promoter element motif for the *CYC1* promoter than “TATTTAAA” (8 bp). Furthermore, “TATTTAAA” (8 bp) may be another distinct gene-specific core promoter element motif, since it was active in the *ADE* promoters but not in the *CYC1* and *HIS3* (e.g., aforementioned “R15/CACCGCTATTTAAATCCC”) promoters. Therefore, it is likely that not only the sequence but also the length of the core promoter element(s) play a critical role in their gene-specific function, as evidenced by the relationship between TATTTAAA (8 bp) and TATTTAAAA (9 bp), or that between TATAWAD (7 bp) and TATAWAWR (8 bp).

## Conclusion

In the present study, we identified a large number of sequences that could function as the *CYC1*-TATAα element by using a novel reporter assay system. This assay system is convenient, sensitive, and reliable, as shown by its ability to isolate all active 18 variants among the 23 specifically mutated *CYC1*-TATAα elements [54] (S4–S7 Tables). Our results showed that TATAWAD is a functional consensus TATA element motif for the *CYC1* promoter and suggested that several other sequence motifs function in a gene-specific manner. The isolated sequences were classified tentatively into nine groups; however, they showed considerable variation in transcriptional activity even within a single class (Fig 5A, S2–S4 Figs). To further delineate the consensus sequence(s) for the functional core promoter element motif(s), more detailed statistical and/or mutational analyses need to be performed in a large number of sequences in each class. In addition, a similar screen may need to be conducted more extensively and systematically in a wide variety of promoters. Nevertheless, we believe that these experimental approaches could provide valuable information for understanding not only the structures of the core promoter element(s), but also the mechanisms underlying the functional compatibilities between the UAS and the core promoter.

## Supporting Information

S1 Fig Transcription from the *CYC1* promoter at the *VTC1* locus is Taf1p-dependent. **A**. Northern blot analyses to examine the expression of the reporter (*VTC1*) or control gene (*SCR1*) in *TAF1* or *taf1-N568Δ* strains carrying the reporter driven by wild-type (β, α, wt, wt at site #1, 2, 3, 4, respectively) or variously mutated *CYC1* promoters as indicated at the top. The details of the promoter sequences tested here were shown in Fig 2A. Total RNA (20 μg) was isolated from these strains 2 hours after temperature shift to 37°C or continuously cultured at 25°C over the same time period in rich media (YPD), and blotted onto the membrane and hybridized with the gene-specific probes indicated at the right. **B**. The raw data shown in **A** were quantified and presented graphically. Values for each *VTC1* transcript were normalized by *SCR1* (pol III transcript) and presented as relative to those derived from the *TAF1* strain carrying the reporter driven by the wild-type *CYC1* promoter (β, α, wt, wt) and cultured at 25°C or 37°C (i.e., the values at 1^st^ or 11^th^ lane are set as 1 for 25°C or 37°C, respectively). To emphasize the contribution of the TATA element(s) at site #1 or #2 to transcriptional activity, the background levels represented by the (m, m, m, m) promoter (i.e., 5^th^, 10^th^, 15^th^ and 20^th^ lanes in each set) are colored in gray.
(EPS)

S2 Fig Enlarged Figures of the right panel in Fig 5A for readability of each sequence of the isolated clones (class I, II and III). The scales at the horizontal axis are 10 (clone number) or 0.1 (normalized value) per division, respectively. **A**. Class I (TATAWAWR). **B**. Class II (TATAWAD). **C**. Class III (GAAAA). The GAAAA-containing sequences were searched for the isolated clones by using two distinct frames; one is Nx9 (9 bp; TATATAAAA, upper panel) and the other is G/Nx9 (10 bp; GTATATAAAA, lower panel). Note that only the former is shown in Fig 5A. The boundary between the clones isolated more than twice and those isolated only once is marked with a closed triangle as described in Fig 5A.
(EPS)

S3 Fig Enlarged Figures of the right panel in Fig 5A for readability of each sequence of the isolated clones (class IV and V). The scales and closed triangles are as described in S2 Fig. **A**. Class IV (TATAWKW). **B**. Class V (TTAAAW).
(EPS)

S4 Fig Enlarged Figures of the right panel in Fig 5A for readability of each sequence of the isolated clones (class VI, VII, VIII and IX). The scales are as described in S2 Fig. Only reproducible clones isolated more than twice are shown in this figure. **A**. Class VI (Wx6). **B**. Class VII (TATATCWD). **C**. Class VIII (Wx5*). **D**. Class IX (others).
(EPS)

S5 Fig Classification and comparison of the clones/sequences isolated by the screen with the sequences utilized as the PIC assembly sites in the Taf1p-enriched or Taf1p-depleted promoters. **A**. Summarized table for classification of the clones/sequences isolated by the screen and comparison of their sequences with those of the PIC assembly sites on the genome as described in Fig 5B, except that the latter (indicated as “all”) was further classified into two groups (indicated as “Taf1p-enriched” and “Taf1p-depleted”) [27]. The significance of the differences between the screen and genome-wide “isolatable” data for each class was evaluated by using the Z test. The calculated p-values are shown in the 10^th^, 14^th^ and 18^th^ columns, respectively. Abbreviations are the same as those described in Fig 5B except that pro # = promoter # and N/A = not applicable. **B**. The percentages of the “normalized” value (*CYC1*) and those of the “promoter #” (genome-wide) were presented graphically as described in Fig 5C.
(EPS)

S6 Fig Comparison of the normalized* isolation frequencies of the individual sequences belonging to class I (TATAWAWR) or II (TATAWAD) that were isolated by the screen with the utilization frequencies as the PIC assembly sites on the genome. The results were summarized in a table (**A**) or four graphs (**B**). In both **A** and **B**, the sequences are aligned according to the promoter number in the “all” category of the PIC assembly sites. Note that the normalized* value in this figure is not the same as the normalized value in Fig 5 and S2–S5 Figs. Each sequence (8 bp: TATAWAWT #1–8 & TATAWAD #1–16) in the list as summarized in **A** includes 1–6 distinct isolated sequences (9 bp). For instance, TATATATA (TATAWAWR #1/ 107 clones) includes six distinct sequences (TATATATAA/ 61 clones, TATATATAT/ 3 clones, TATATATAG/ 8 clones, TATATATAC/ 3 clones, ATATATATA/ 15 clones, TTATATATA/ 17 clones), whereas TATAAATG (TATAWAWR #8/ 20 clones) contains only one sequence (TATAAATGA/ 20 clones) (S7 Table). The normalized* value (e.g., TATATATA: 0.74, TATAAATG: 0.58) was calculated by dividing the total number of isolated clones (9 bp) containing a given sequence (8 bp) (e.g., TATATATA: 61+3+8+3+15+17 = 107, TATAAATG: 20) by the expected number of the clones containing the same 8 bp sequence that exist in the libraries (e.g., TATATATA: 75.625+13.75+13.75+13.75+13.75+13.75 = 144.375, TATAAATG: 34.375, as listed in S7 Table), not by summing up the normalized value determined individually for each sequence (9 bp) as described in the legend of Fig 5 (e.g., TATATATA: 61/75.625+3/13.75+8/13.75+3/13.75+15/13.75+17/13.75 = 4.15, TATAAATG: 20/34.375 = 0.58). The normalized value (e.g., TATATATA: 4.15, TATAAATG: 0.58) deviates significantly from the normalized* value that does not exceed 1.0 theoretically (e.g., TATATATA: 0.74, TATAAATG: 0.58), especially when target sequence(s) are generated from multiple libraries. We suppose that the normalized value is more appropriate for the purpose of Fig 5 and S2–S5 Figs than the normalized* value, since the integral of the values that have been normalized individually for each sequence is more informative for the comparison between several classes, each of which includes a huge number of sequences. On the contrary, the normalized* value is more appropriate for the purpose of this figure than the normalized value, since the latter is affected significantly by the number of isolated sequences (9 bp) included in each target sequence (8 bp). Such artificial effects derived from the library construction strategy need to be minimized for the comparison attempted here. Thus, we exploit the normalized* value in this figure, in which all isolated sequences (9 bp) containing the same 8 bp sequence were treated evenly and subjected as a set to the calculation for normalization.
(EPS)

S7 Fig Sequences of the *ADE5*,*7* (A) and *ADE6* (B) promoters. The TATTTAAA sequence and its mutated derivative (mut: CGCCCGGG) are marked with a thick black line. The major transcriptional start sites (TSSs) determined by primer extension analyses as described in Fig 6C are indicated below the sequence with a black arrow, while the TSSs determined in a previous study [75] are indicated above the sequence with a gray arrow as a reference. The initiation codon of each gene is marked with an open square along with the number of +1 (A of ATG as +1).
(EPS)

S1 Table *Saccharomyces cerevisiae* strains used in this study. (DOC)

S2 Table Oligonucleotides used in this study. (DOC)

S3 Table PCR primers used for the construction of yeast strains in this study. (XLSX)

S4 Table Promoter activities of the clones isolated from the libraries of frame 1–6 & 9. (XLSX)

S5 Table Promoter activities of the clones isolated from the library of frame 7. (XLSX)

S6 Table Promoter activities of the clones isolated from the library of frame 8. (XLSX)

S7 Table Normalized values of the clones isolated from the libraries of frame 1–6 & 9. (XLS)
