# Supplementary material for: A Random Screen Using a Novel Reporter Assay System Reveals a Set of Sequences That Are Preferred as the TATA or TATA-Like Elements in the CYC1 Promoter of Saccharomyces cerevisiae
Source: PLoS One. 2015 Jun 5;10(6):e0129357. doi: 10.1371/journal.pone.0129357 (PMC4457894; doi:10.1371/journal.pone.0129357)
Supplement: S1 Table — (DOC) [file pone.0129357.s008.doc]

S1 Table. *S. cerevisiae* strains used in this study (K. Watanabe et al.)

---------------------------------------------------------------------------------------------------------------------------------------------------------------------------

Strain Genotype Source

---------------------------------------------------------------------------------------------------------------------------------------------------------------------------

BY4741 *MATa his3Δ1 leu2Δ0 met15Δ0 ura3Δ0* Euroscarf

Y00212 *MATa his3Δ1 leu2Δ0 met15Δ0 ura3Δ0 vtc1Δ0::kanMX4* Euroscarf

Y22.1 *MATα ura3-52 trp1-63 leu2-3,112 Δtaf1::hisG* pYN1/*TAF1* T. Kokubo (1998)

YTK2741 *MATα ura3-52 trp1-63 leu2-3,112 Δtaf1::hisG* pM1169/*TAF1* S. Takahata (2004)

YTK3778 *MATα ura3-52 trp1-63 leu2-3,112 Δtaf1::hisG* pM1746/*taf1-N568Δ* this study

YTK6352 *MATα his3Δ1 leu2Δ0 lys2Δ0 ura3Δ0 TAF1::kanMX6* #1 promoter-*VTC1::His3MX6* S. Ki (2006)

YTK6353 *MATα his3Δ1 leu2Δ0 lys2Δ0 ura3Δ0 TAF1::kanMX6* #2 promoter-*VTC1::His3MX6* S. Ki (2006)

YTK6356 *MATα his3Δ1 leu2Δ0 lys2Δ0 ura3Δ0 TAF1::kanMX6* #3 promoter-*VTC1::His3MX6* S. Ki (2006)

YTK6359 *MATα his3Δ1 leu2Δ0 lys2Δ0 ura3Δ0 TAF1::kanMX6* #4 promoter-*VTC1::His3MX6* S. Ki (2006)

YTK6362 *MATα his3Δ1 leu2Δ0 lys2Δ0 ura3Δ0 TAF1::kanMX6* #5 promoter-*VTC1::His3MX6* S. Ki (2006)

YTK6365 *MATα his3Δ1 leu2Δ0 lys2Δ0 ura3Δ0 TAF1::kanMX6* #6 promoter-*VTC1::His3MX6* S. Ki (2006)

YTK7548 *MATa his3Δ1 leu2Δ0 met15Δ0 ura3Δ0 CYC1* promoter-*VTC1::His3MX6* this study

YTK7550 *MATa his3Δ1 leu2Δ0 met15Δ0 ura3Δ0 CYC1* promoter [#3 & 4 mut]-*VTC1::His3MX6* this study

YTK7554 *MATa his3Δ1 leu2Δ0 met15Δ0 ura3Δ0 CYC1* promoter [#2, 3 & 4 mut]-*VTC1::His3MX6* this study

YTK7558 *MATa his3Δ1 leu2Δ0 met15Δ0 ura3Δ0 CYC1* promoter [#1, 2, 3 & 4 mut]-*VTC1::His3MX6* this study

YTK7560 *MATa his3Δ1 leu2Δ0 met15Δ0 ura3Δ0 CYC1* promoter [#1, 3 & 4 mut]-*VTC1::His3MX6* this study

YTK7563 *MATa his3Δ1 leu2Δ0 met15Δ0 ura3Δ0 CYC1* promoter [#1α, 2α, 3 & 4 mut]-*VTC1::His3MX6* this study

YTK7565 *MATa his3Δ1 leu2Δ0 met15Δ0 ura3Δ0 CYC1* promoter [#1β, 2β, 3 & 4 mut]-*VTC1::His3MX6* this study

YTK7567 *MATa his3Δ1 leu2Δ0 met15Δ0 ura3Δ0 CYC1* promoter [#1α, 2α, 3 & 4 mut]-*VTC1::His3MX6* this study

YTK7569 *MATa his3Δ1 leu2Δ0 met15Δ0 ura3Δ0 CYC1* promoter [#1β, 2β, 3 & 4 mut]-*VTC1::His3MX6* this study

YTK7796 *MATa his3Δ1 leu2Δ0 met15Δ0 ura3Δ0 CYC1* promoter [#1α, 2β, 3 & 4 mut]-*VTC1::His3MX6* this study

YTK7798 *MATa his3Δ1 leu2Δ0 met15Δ0 ura3Δ0 CYC1* promoter [#1α, 2, 3 & 4 mut]-*VTC1::His3MX6* this study

YTK7800 *MATa his3Δ1 leu2Δ0 met15Δ0 ura3Δ0 CYC1* promoter [#1mut, 2β, 3 & 4 mut]-*VTC1::His3MX6* this study

YTK16302 *MATa his3Δ1 leu2Δ0 met15Δ0 ura3Δ0 CYC1* promoter [#1α, 2β, 3 & 4 mut]-*VTC1::His3MX6* this study

YTK16303 *MATa his3Δ1 leu2Δ0 met15Δ0 ura3Δ0 CYC1* promoter [#1α, 2, 3 & 4 mut]-*VTC1::His3MX6* this study

YTK16304 *MATa his3Δ1 leu2Δ0 met15Δ0 ura3Δ0 CYC1* promoter [#1mut, 2β, 3 & 4 mut]-*VTC1::His3MX6* this study

YTK16396 *MATα ura3-52 trp1-63 leu2-3,112 Δtaf1::hisG CYC1* promoter [#1, 3 & 4 mut]-*VTC1::LEU2* pM1169/*TAF1* this study

YTK16397 *MATα ura3-52 trp1-63 leu2-3,112 Δtaf1::hisG CYC1* promoter [#1, 3 & 4 mut]-*VTC1::LEU2* pM1746/*taf1-N568Δ* this study

YTK16398 *MATα ura3-52 trp1-63 leu2-3,112 Δtaf1::hisG CYC1* promoter [#1, 2, 3 & 4 mut]-*VTC1::LEU2* pM1169/*TAF1* this study

YTK16399 *MATα ura3-52 trp1-63 leu2-3,112 Δtaf1::hisG CYC1* promoter [#1, 2, 3 & 4 mut]-*VTC1::LEU2* pM1746/*taf1-N568Δ* this study

YTK16418 *MATa his3Δ1 leu2Δ0 met15Δ0 ura3Δ0 ADE5,7* promoter [wt:TATTTAAA]-*VTC1::His3MX6* this study

YTK16419 *MATa his3Δ1 leu2Δ0 met15Δ0 ura3Δ0 ADE5,7* promoter [mut:CGCCCGGG]-*VTC1::His3MX6* this study

YTK16421 *MATa his3Δ1 leu2Δ0 met15Δ0 ura3Δ0 ADE6* promoter [wt:TATTTAAA]-*VTC1::His3MX6* this study

YTK16422 *MATa his3Δ1 leu2Δ0 met15Δ0 ura3Δ0 ADE6* promoter [mut:CGCCCGGG]-*VTC1::His3MX6* this study

YTK16455 *MATα ura3-52 trp1-63 leu2-3,112 Δtaf1::hisG CYC1* promoter-*VTC1::LEU2* pM1169/*TAF1* this study

YTK16456 *MATα ura3-52 trp1-63 leu2-3,112 Δtaf1::hisG CYC1* promoter-*VTC1::LEU2* pM1746/*taf1-N568Δ* this study

YTK16457 *MATα ura3-52 trp1-63 leu2-3,112 Δtaf1::hisG CYC1* promoter [#3 & 4 mut]-*VTC1::LEU2* pM1169/*TAF1* this study

YTK16458 *MATα ura3-52 trp1-63 leu2-3,112 Δtaf1::hisG CYC1* promoter [#3 & 4 mut]-*VTC1::LEU2* pM1746/*taf1-N568Δ* this study

YTK16459 *MATα ura3-52 trp1-63 leu2-3,112 Δtaf1::hisG CYC1* promoter [#2, 3 & 4 mut]-*VTC1::LEU2* pM1169/*TAF1* this study

YTK16460 *MATα ura3-52 trp1-63 leu2-3,112 Δtaf1::hisG CYC1* promoter [#2, 3 & 4 mut]-*VTC1::LEU2* pM1746/*taf1-N568Δ* this study

---------------------------------------------------------------------------------------------------------------------------------------------------------------------------
