# Supplementary material for: A Random Screen Using a Novel Reporter Assay System Reveals a Set of Sequences That Are Preferred as the TATA or TATA-Like Elements in the CYC1 Promoter of Saccharomyces cerevisiae
Source: PLoS One. 2015 Jun 5;10(6):e0129357. doi: 10.1371/journal.pone.0129357 (PMC4457894; doi:10.1371/journal.pone.0129357)
Supplement: S2 Table — (DOC) [file pone.0129357.s009.doc]

S2 Table Oligonucleotides used in this study (K. Watanabe et al.)

---------------------------------------------------------------------------------------------------------------------------------------------

Number Sequence

---------------------------------------------------------------------------------------------------------------------------------------------

TK176 5'-GCCAATAAATTAATTTATTATCGGAAAGCC-3'

TK2496 5'-AAATAGATATTAGCACGTGTCTCGG-3'

TK4283 5'-TTTGTGCGTAACCCACGCTTACGATATTGGAATTACAATT-3'

TK6582 5'-GTCGACGGATCCCCGGGAATTCGATCCGCAGGCTAACCGGAA-3'

TK7872 5'-TCGGATAGAATGGCAAGAGC-3'

TK7873 5'-TCTGGCGTAGTGTTTAATCG-3'

TK7875 5'-AAAAAGCACAACAAAGTGGG-3'

TK8251 5'-TCGTGTGGGCAAAGCGATCT-3'

TK8260 5'-CGGTATCCGCTAATAGAGAG-3'

TK8297 5'-ATACGATTAAACACTACGCCAGATTTCCACAATGAATTCGAGCTCGTTTAAAC-3'

TK8379 5'-ATCAAACCGATTTGGCGCAAG-3'

TK8380 5'-AGAGTCTGCACCTGGCCTTCT-3'

TK8381 5'-TGTAATGAATCACAAGAACGT-3'

TK8382 5'-ATTAGGGCGTTAACTGGTTTT-3'

TK9013 5'-TAACTTAGTGTTAGCGTCATT-3'

TK9030 5'-ATGTCTTCAGCACCATTATTACAA-3'

TK9044 5'-TGTAAAGTTCAACCACGACA-3'

TK9507 5'-AGGCTGTAATGGCTTTCT-3'

TK9613 5'-TCTTTGTAATAATGGTGCTGAAGAC-3'

TK10036 5'-GAATTCCCGGGGATCCGTCGACTGAATCTAAAATTCCCGGGA-3'

TK10037 5'-TTTTCCCAGGTGTTCTTTGTAATAATGGTGCTGAAGACATTATTAATTTAGTGTGTGTAT-3'

TK10038 5'-TAGAGAAAAGAAGAAAACAAG-3'

TK10081 5'-CCACAATGTGCGAGTAAATCC-3'

TK10254 5'-AACTCTTGTTTTCTTCTTTTCTCTAGGCCTTCTTTCCTTGCGCATTAGGACCTTTGCAGCATA-3'

TK10255 5'-TGCCTGTATGTGTCAGCACTA-3'

TK10256 5'-ACTTTAGTGCTGACACATACAGGCATACGCGTATGTGTGCGACGACACATG-3'

TK10257 5'-CATACAGAGCACATGCATGCC-3'

TK10258 5'-ATATGGCATGCATGTGCTCTGTATGTAGCGCAAACTCTTGTTTTCTTCTTT-3'

TK10261 5'-ATATGGCATGCATGTGCTCTGTATGWWWWWWWWACTCTTGTTTTCTTCTTTTC-3'

TK10262 5'-ATATGGCATGCATGTGCTCTGTATGTATANNNNACTCTTGTTTTCTTCTTTTC-3'

TK10263 5'-GTCGACGGATCCCCGGGAATTCCGGATCCCCGGGTTAATTAA-3'

TK10267 5'-GCAGAGGAGAGTTATCACTCCTTCCATCCT-3'

TK10696 5'-ACTTTAGTGCTGACACATACAGGCATATATAAATGTGTGCGACGACACATGAT-3'

TK10697 5'-ATATGGCATGCATGTGCTCTGTATGTATATATAACTCTTGTTTTCTTCTTTTC-3'

TK10709 5'-ACTTTAGTGCTGACACATACAGGCATATATAAAAGTGTGCGACGACACATGAT-3'

TK10710 5'-ATATGGCATGCATGTGCTCTGTATGTATATATATATCTTGTTTTCTTCTTTTC-3'

TK11272 5'-ATATGGCATGCATGTGCTCTGTATGNNNNTAAAACTCTTGTTTTCTTCT-3'

TK11835 5'-ATATGGCATGCATGTGCTCTGTATGTANNNNAAACTCTTGTTTTCTTCTTTTC-3'

TK11836 5'-ATATGGCATGCATGTGCTCTGTATGTATATANNNNTCTTGTTTTCTTCTTTTC-3'

TK11910 5'-ATATGGCATGCATGTGCTCTGTATGTATATAANNNNCTTGTTTTCTTCTTTTC-3'

TK12044 5'-ATATGGCATGCATGTGCTCTGTATGTNNNNAAAACTCTTGTTTTCTTCTTTTC-3'

TK12045 5'-ATATGGCATGCATGTGCTCTGTATGTATNNNNAACTCTTGTTTTCTTCTTTTC-3'

TK12046 5'-ATATGGCATGCATGTGCTCTGTATGTATATNNNNCTCTTGTTTTCTTCTTTTC-3'

TK12262 5'-ATACGATTAAACACTACGCCAGATTTCCACAATGAGCTCGCTGTGAAGATCCC-3'

TK12877 5'-CAGCTTGGTGACAAGAACGTG-3'

TK12878 5'-TAGATTATCGACTCTGAACTGAGA-3'

TK12306 5'-CTATGTTCAGAGGCAAGTGC-3'

TK12310 5'-TAATTGGCTGTCCCTGCCTC-3'

TK12324 5'-GAATTCCCGGGGATCCGTCGACTTACTACAACCCCACACAGG-3'

TK12325 5'-GAATTCCCGGGGATCCGTCGACATATCATATTAAATTGGATT-3'

TK12326 5'-TCGAGGCAGGGACAGCCAATTACCCGGGCGCGTATGTCAAGGTTTTCAAA-3'

TK12327 5'-TTGCACTTGCCTCTGAACATAGCCCGGGCGCCAAGTTCAAGCCCATCG-3'

TK12361 5'-TTTTCCCAGGTGTTCTTTGTAATAATGGTGCTGAAGACATTGTGAGGGGAGGGAGAATGG-3'

TK12362 5'-TTTTCCCAGGTGTTCTTTGTAATAATGGTGCTGAAGACATTGAACTTGACTTCTTTTGTT-3'

---------------------------------------------------------------------------------------------------------------------------------------------
